# Clinical and serological characteristics of peripheral neuropathy in patients with primary Sjögren's syndrome

**DOI:** 10.3389/fneur.2026.1800598

**Published:** 2026-04-30

**Authors:** Wenjing Yue, Yusheng Lv, Dongxia Liu, Hongsheng Sun

**Affiliations:** Department of Rheumatology and Immunology, Shandong Provincial Hospital Affiliated to Shandong First Medical University, Jinan, Shandong, China

**Keywords:** corticosteroids, immunological profile, peripheral neuropathy, primary Sjögren's syndrome, sensorimotor neuropathy

## Abstract

**Objective:**

To investigate the clinical features and serological characteristics of patients with primary Sjögren's syndrome (pSS) complicated with sensorimotor neuropathy (SMN), and to provide a reference for the clinical diagnosis, disease assessment and treatment of this condition.

**Methods:**

A retrospective analysis was conducted on a cohort of 92 patients diagnosed with pSS who were admitted to the Department of Rheumatology and Immunology at Shandong Provincial Hospital between 2016 and 2024. According to the presence and type of peripheral nerve lesions, we divided the patients into three groups: the sensorimotor neuropathy group (SMN group, *n* = 13), the sensory neuropathy group (SN group, *n* = 12), and the non-peripheral neuropathy group (nPN group, *n* = 67). We collected and compared clinical and laboratory data among the three groups and analyzed the neuroelectrophysiological characteristics in the SMN and SN groups.

**Results:**

Among 92 patients with pSS, 25 (27%) exhibited peripheral nervous system involvement, of which 13 (14%) had SMN. Compared with the nPN group, the SMN group showed lower rates of positivity for SSA antibody, SSB antibody, and rheumatoid factor. Additionally, the SMN group had higher median white blood cell count and neutrophil count scores than the nPN group. When compared with the SN group, the SMN group had a higher median age and a shorter disease duration. The average white blood cell count and median neutrophil count of the SMN group were both higher than those of the SN group.

**Conclusions:**

SMN is a common form of peripheral nerve injury observed in pSS patients. These SMN patients display a potentially distinct clinical and serological profile, characterized by low positive rates of anti-SSA/SSB antibodies and rheumatoid factor, as well as elevated peripheral blood inflammatory markers. As this was a single-center, small-sample retrospective observational study, further validation by large-scale prospective studies is warranted.

## 1 Introduction

Primary Sjögren's syndrome (pSS) is a systemic autoimmune disorder characterized by lymphocytic infiltration, primarily affecting exocrine glands such as the lacrimal and salivary glands. In addition, it may also involve various extraglandular organs, particularly the nervous system, lungs, and kidneys. Among these, neurological involvement is increasingly recognized and emphasized due to its complex clinical manifestations and profound impact on patients' quality of life (1–3).

Epidemiological studies indicate that the prevalence of neurological involvement in patients with pSS ranges from 10% to 50%, with significant variability across different reports (4, 5). Such differences may be related to the selection of study populations, variations in diagnostic criteria, and the sensitivity of detection methods. Neurological involvement in pSS can be divided into central and peripheral nervous system involvement, with the latter being more common, accounting for more than 70% of pSS-related neurological manifestations. The clinical manifestations of pSS-associated peripheral neuropathy are complex and diverse. According to the type and functional characteristics of the affected nerve fibers, they can be mainly summarized as follows: sensorimotor neuropathy (SMN), sensory neuropathy (SN), and autonomic dysfunction (6). Among these, SMN is the most prevalent subtype. Clinically, it is characterized by paresthesia, symmetrically distributed muscle weakness ranging from mild impairment to severe disability requiring wheelchair dependence or bed rest, as well as diminished or absent deep tendon reflexes (7, 8). Nerve conduction studies (NCS) commonly demonstrate axonal involvement affecting both motor and sensory fibers. Neuropathic pain and motor dysfunction significantly affect patients' quality of life (9).

Currently, there is limited research on pSS complicated by SMN, and no consensus has been reached regarding its underlying pathophysiological mechanisms and optimal diagnostic and therapeutic strategies. By analyzing the clinical, serological, and neuroelectrophysiological characteristics of pSS patients complicated with SMN, this study aims to improve clinical understanding of the disease and provide a reference for clinical diagnosis and treatment.

## 2 Materials and methods

### 2.1 Patients

A retrospective analysis was conducted on the clinical data of 92 patients with pSS who were hospitalized in the Rheumatology and Immunology Department of our hospital between 2016 and 2024. According to clinical manifestations and nerve fiber involvement, patients were divided into the sensorimotor neuropathy group (SMN group, *n* = 13), the sensory neuropathy group (SN group, *n* = 12), and the non-peripheral neuropathy group (nPN group, *n* = 67). The grouping criteria were as follows: (a) SMN group: patients presented with definite sensory abnormalities such as limb numbness, tingling, hypoesthesia and hyperesthesia accompanied by motor dysfunction, and neuroelectrophysiological examination indicated simultaneous involvement of both sensory and motor nerves; (b) SN group: patients only showed sensory abnormalities without any manifestations related to motor dysfunction such as limb weakness, and neuroelectrophysiological examination revealed only sensory nerve involvement; (c) nPN group: patients had no clinical symptoms associated with peripheral nerve involvement, and all indicators of neuroelectrophysiological examination were within the normal reference range, suggesting no peripheral nerve involvement (10–12).

All patients met the 2012 diagnostic criteria for pSS published by Sjögren's International Collaborative Clinical Alliance (SICCA) (13). Patients with overlapping syndromes (including but not limited to rheumatoid arthritis, systemic lupus erythematosus, or systemic sclerosis) were excluded from the study. In addition, other potential causes of peripheral neuropathy were systematically evaluated and excluded, including paraneoplastic syndrome, alcoholism, diabetes mellitus, drug-related neuropathy, hypothyroidism, and amyloidosis. All patients were evaluated by two rheumatology and immunology specialists.

### 2.2 Neurological evaluation

The diagnosis of peripheral neuropathy was established based on objective clinical signs combined with electrophysiological examination results. NCS and electromyography were performed. For motor nerve conduction, the examined nerves mainly included the median nerve, ulnar nerve, tibial nerve, and common peroneal nerve. For sensory nerve conduction, the examined nerves mainly included the median nerve, ulnar nerve, sural nerve, and superficial peroneal nerve. Nerve conduction velocity, amplitude, latency, distance, and F-wave parameters were evaluated. During electromyography, the examined muscles mainly included the quadriceps femoris, gastrocnemius, tibialis anterior, biceps brachii, deltoid, and abductor pollicis brevis. Spontaneous activities such as fibrillation potentials, positive sharp waves, and myotonic discharges were recorded, as well as the amplitude, duration, and polyphasicity of motor unit potentials.

### 2.3 Laboratory data

Routine laboratory test results and immunological parameters were recorded for all patients. Anti-Ro/SSA and anti-La/SSB antibodies were detected by enzyme-linked immunosorbent assay or immunoblotting, rheumatoid factor (RF) was measured by nephelometry, other items included immunoglobulins, complement C3 and C4, and peripheral blood count.

### 2.4 Disease activity

The activity of pSS was evaluated using the European League Against Rheumatism Disease Activity Index (ESSDAI). The ESSDAI comprises 12 domains: “Constitutional, Lymphadenopathy and lymphoma, Glandular, Articular, Muscular, Cutaneous, Pulmonary, Renal, Peripheral nervous system, Central nervous system, Hematological, Biological.”

### 2.5 Statistical analysis

The statistical software SPSS 25.0 for Windows was used to analyze the data. The continuous variables were expressed as mean ± standard deviation (x ± s) or median (1st quartile, 3rd quartile) [*M* (*P*_25_, *P*_75_)], and the categorical variables were expressed as *n* (%). The independent sample *t* test was used for comparison of continuous variables in normal distribution between groups, and Mann Whitney *U* test was used for comparison of continuous variables in non-normal distribution between groups. Categorical variables were analyzed by χ^2^ test. *P* < 0.05 was considered statistically significant.

## 3 Results

### 3.1 Baseline characteristics and subtypes of peripheral neuropathy

A total of 92 patients with pSS were included in this study, including 86 females (93.5%) and 6 males (6.5%), with a male-to-female ratio of 3:43, which is consistent with the epidemiological characteristics of predominant female involvement in pSS. The age ranged from 14 to 84 years, with a mean age of 53.93 ± 14.72 years, a mean onset age of 53.20 ± 14.62 years, and a mean disease duration of 3.44 ± 4.87 years. These findings indicate that the disease mostly affects middle-aged and elderly populations.

**Figure 1:**
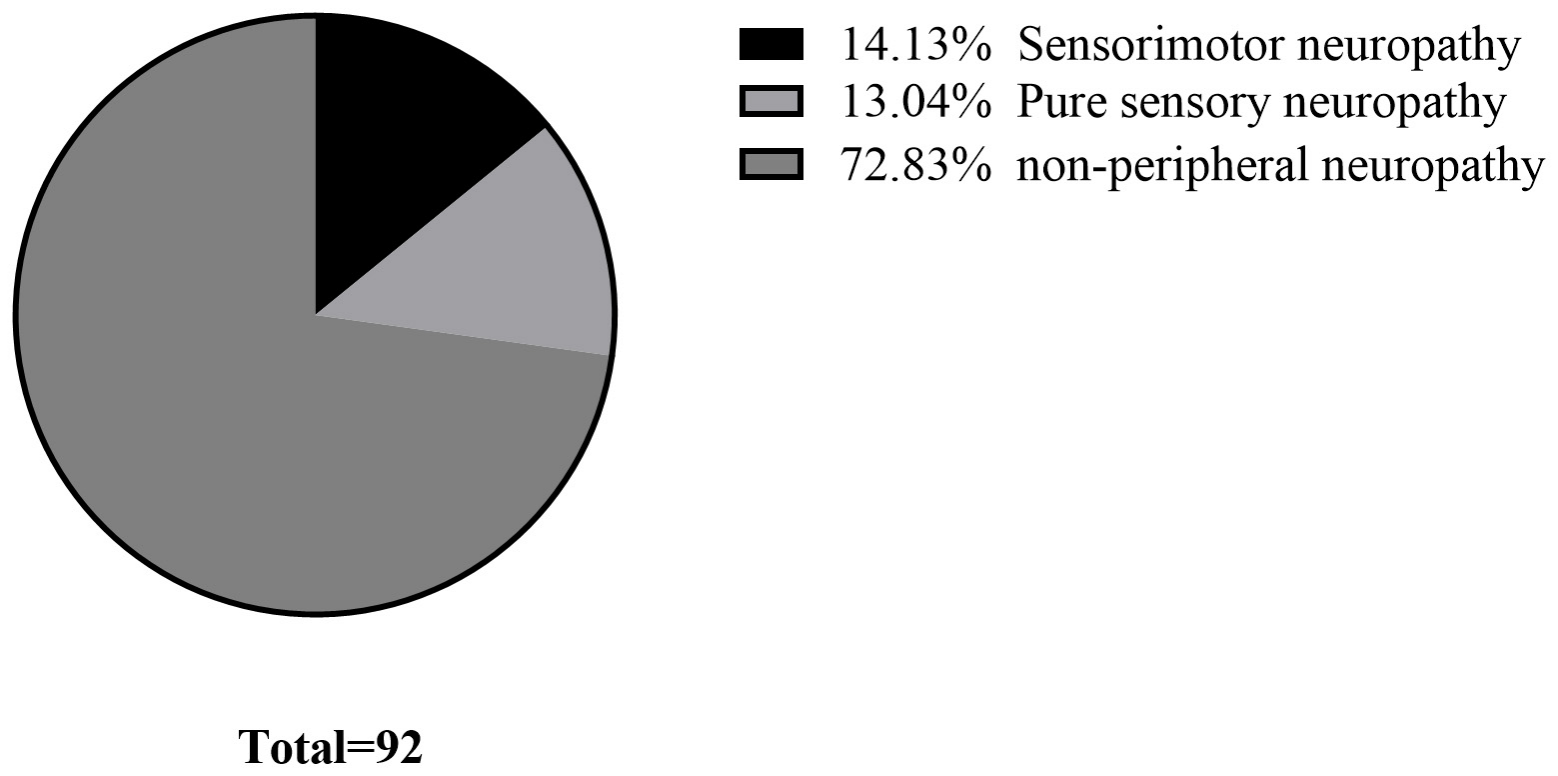
Profile of neuropathy status in pSS (*n* = 92).

All the patients completed nerve conduction studies, 67 patients (72.83%) showed no significant abnormalities. The remaining 25 patients were confirmed to have neuroelectrophysiological abnormalities, such as altered nerve conduction velocity or decreased action potential amplitude. The proportions of the SMN type and SN type were 14.13% and 13.04%, respectively (as shown in Figure 1).

### 3.2 Comparison of clinical and laboratory characteristics between the SMN group and the nPN group

The clinical data of 13 pSS patients with SMN and 67 pSS patients with nPN were analyzed and compared, as shown in Table 1. In the SMN group and the nPN group, the median ages were 60 (56, 63) years and 57 (43, 66) years, respectively (*P* = 0.540). Compared with the nPN group, the SMN group exhibited lower rates of anti-SSA antibody positivity (46.2% vs. 83.3%, *P* = 0.010), anti-SSB antibody positivity (23.1% vs. 53.7%, *P* = 0.043), and RF positivity (9.1% vs. 53.6%, *P* = 0.007). However, the SMN group had higher median white blood cell count (7.58 vs. 4.20, *P* < 0.001), median neutrophil count (4.33 vs. 2.33, *P* = 0.011). No statistically significant differences were observed between the two groups in terms of disease duration, positive rates of anti-ANA antibodies, IgG levels, C3 levels, C4 levels.

**Table 1:** Comparison of basic demographic characteristics and laboratory data between the SMN and nPN groups (*n* = 80).

Variable	SMN (*n* = 13)	nPN (*n* = 67)	*P*-value
Age (years)	60 (56, 63)	57 (43, 66)	0.540
Disease duration (years)	0.83 (0.08, 2.00)	1.00 (0.42, 5.00)	0.102
Positive anti-ANA (%)	12 (92.3%)	65 (97.0%)	0.984
Positive anti-Ro/SSA (%)^a^	6 (46.2%)	55 (83.3%)	**0.010**
Positive anti-La/SSB (%)	3 (23.1%)	36 (53.7%)	**0.043**
Positive RF (%)^b^	1 (9.1%)	30 (53.6%)	**0.007**
IgG (g/L)	15.40 (13.40, 16.90)	18.60 (14.50, 21.70)	0.062
C3 (g/L, mean ± SD)	1.14 ± 0.31	1.02 ± 0.20	0.092
C4 (g/L, mean ± SD)	0.26 ± 0.08	0.22 ± 0.07	0.237
Leukocytes ( × 10^9^/L)	7.58 (5.20, 11.30)	4.20 (3.38, 6.05)	**< 0.001**
Neutrophils ( × 10^9^/L)	4.33 (2.80, 6.04)	2.33 (1.74, 3.55)	**0.011**
Prednisolone (%)	12 (92.3%)	49 (73.1%)	0.258
Mycophenolate mofetil (%)	5 (38.5%)	28 (41.8%)	0.823
Hydroxychloroquine (%)	5 (38.5%)	32 (47.8%)	0.538
Cyclophosphamide (%)	2 (15.4%)	1 (1.5%)	0.067

^a^For anti-Ro/SSA, the denominator was n = 66 in the nPN group due to 1 missing value.

^b^For RF, the denominators were n = 11 (SMN) and n = 56 (nPN) due to missing data.

SMN, sensorimotor neuropathy; nPN, pSS without peripheral nervous system involvement; ANA, Antinuclear antibody; RF, rheumatoid factor; ACA, Anticardiolipin antibody. Bold values indicate statistically significant differences (*P* < 0.05).

### 3.3 Comparison of clinical, laboratory characteristics and ESSDAI systemic involvement between the SMN group and the SN group

As shown in Table 2, compared with the SN group, the SMN group exhibited higher median age (60.00 vs 50.00, *P* = 0.036) and a shorter disease duration (0.83 vs 2.50, *P* = 0.040). Additionally, the SMN group also showed higher mean white blood cell count (8.20 ± 3.30 vs 5.44 ± 1.87, *P* = 0.017) and a higher median neutrophil count (5.23 vs 3.14, *P* = 0.039) compared to the SN group. No significant differences were observed between the two groups in terms of antibody positivity rates (ANA, SSA, SSB, and RF), levels of C3, C4 or the use of medications including prednisone, mycophenolate mofetil, and hydroxychloroquine.

**Table 2:** Comparison of baseline demographic characteristics and laboratory parameters between the SMN and SN groups (*n* = 25).

Variable	SMN (*n* = 13)	SN (*n* = 12)	*P*-value
Age (years)	60.0 (56.0, 63.0)	50.00 (36.50, 55.50)	**0.036**
Disease duration (years)	0.83 (0.08, 2.00)	2.50 (1.00, 5.75)	**0.040**
Positive anti-ANA (%)	12 (92.3%)	12 (100.0%)	>0.999
Positive anti-Ro/SSA (%)	6 (46.2%)	10 (83.3%)	0.097
Positive anti-La/SSB (%)	3 (23.1%)	7 (58.3%)	0.111
Positive RF (%)^a^	1 (9.1%)	5 (45.5%)	0.149
Positive anti- ACA (%)^b^	4 (36.4%)	4 (33.3%)	>0.999
C3 (g/L, mean ± SD)	1.14 ± 0.31	0.98 ± 0.17	0.136
C4 (g/L, mean ± SD)	0.26 ± 0.08	0.21 ± 0.09	0.214
Leukocytes ( × 10^9^/L mean ± SD)	8.20 ± 3.30	5.44 ± 1.87	**0.017**
Neutrophils ( × 10^9^/L)	5.23 (3.32, 8.58)	3.14 (2.12, 4.23)	**0.039**
Prednisolone (%)	12 (92.3%)	10 (83.3%)	0.593
Mycophenolate mofetil (%)	5 (38.5%)	7 (58.3%)	0.434
Hydroxychloroquine (%)	5 (38.5%)	9 (75.0%)	0.111
Cyclophosphamide (%)	2 (15.4%)	0 (0.0%)	0.480

^a^For RF, denominator n = 11 in both groups due to missing data.

^b^For anti-ACA, denominator n = 11 in the SMN group due to 2 missing values.

SMN, sensorimotor neuropathy; SN, sensory neuropathy; ANA, Antinuclear antibody; RF, rheumatoid factor; ACA, Anticardiolipin antibody. Bold values indicate statistically significant differences (*P* < 0.05).

**Figure 2:**
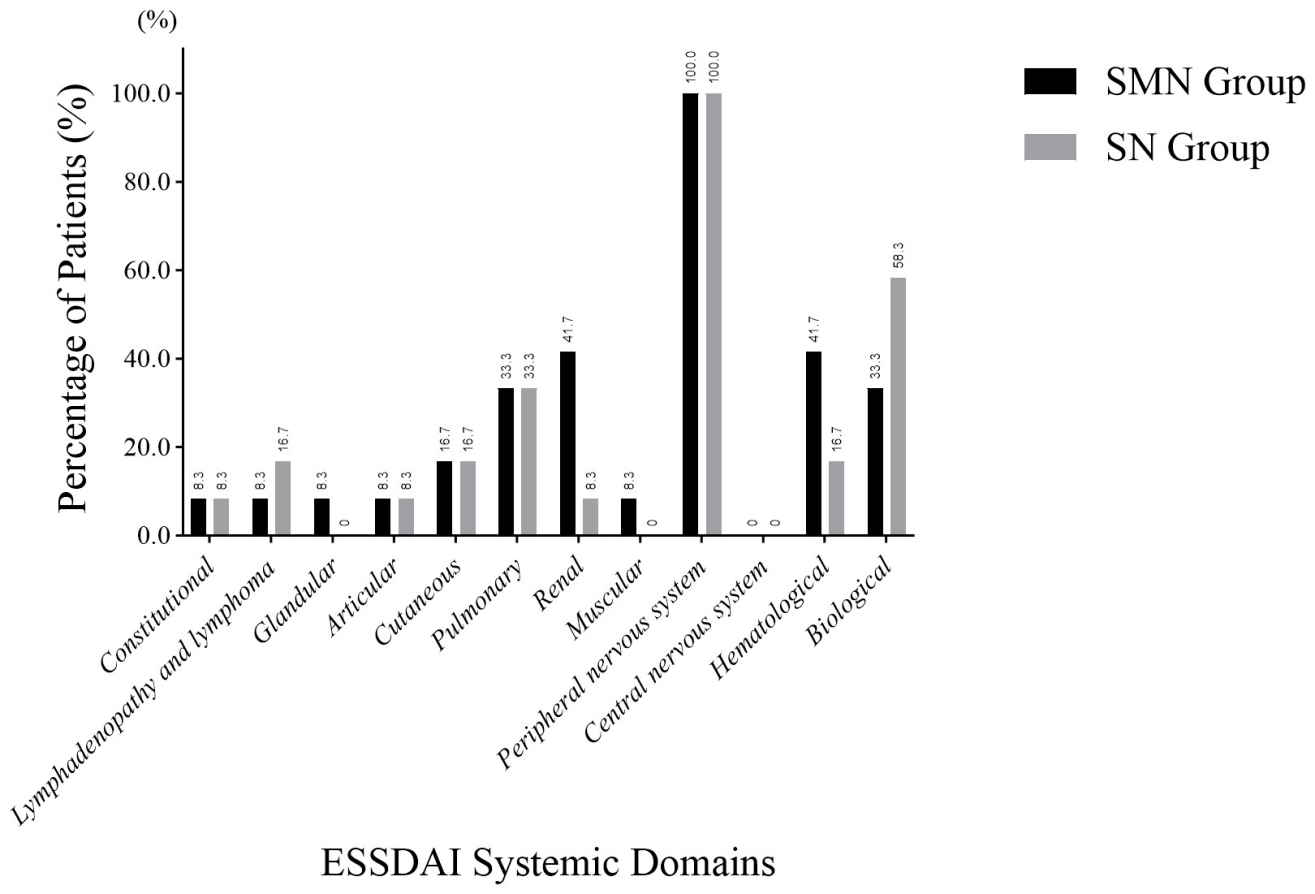
Comparison of the involvement of other systems in the ESSDAI score between the SMN group and the SN group (*n* = 24). Due to unclear documentation of specific ESSDAI domain involvement in some cases, the effective sample size of the SMN group was 12. SMN, sensorimotor neuropathy; SN, sensory neuropathy.

The overall pattern of systemic involvement based on ESSDAI was comparable between the two groups. Due to unclear documentation of specific ESSDAI domain involvement in some cases, the effective sample size of the SMN group was 12. The SMN group exhibited higher rates of renal (41.7% vs 8.3%) and hematological involvement (41.7% vs 16.7%). However, none of these differences reached statistical significance (*P* >0.05). The prevalence of other ESSDAI domains was low and comparable between groups. These findings are illustrated in Figure 2.

### 3.4 Analysis of neuroelectrophysiological characteristics in patients between SMN group and SN group

**Figure 3:**
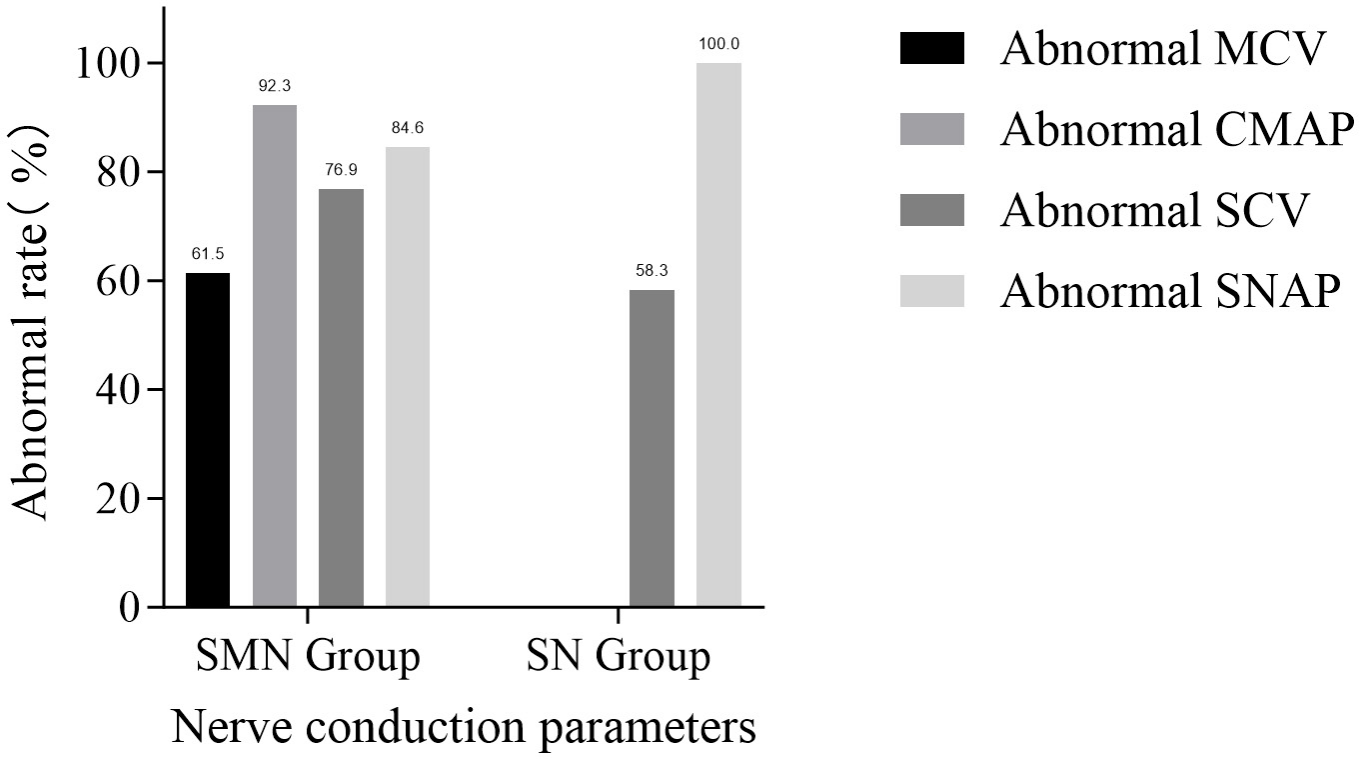
Comparison of abnormal rate of nerve conduction parameters between the SMN group and the SN group (*n* = 25). SMN, sensorimotor neuropathy; SN, sensory neuropathy; MCV, motor nerve conduction velocity; CMAP, compound muscle action potential; SCV, sensory nerve conduction velocity; SNAP, sensory nerve action potential.

This study observed and summarized the abnormal rates of nerve conduction parameters in patients from the SMN group and the SN group (Figure 3). In the SMN group, abnormal motor nerve conduction velocity (MCV) was found in 8 cases (61.5%), abnormal compound muscle action potential (CMAP) in 12 cases (92.3%), abnormal sensory nerve conduction velocity (SCV) in 10 cases (76.9%), and abnormal sensory nerve action potential (SNAP) in 11 cases (84.6%). In the SN group, motor nerve involvement was excluded according to the grouping definition; therefore, only sensory nerve conduction parameters were analyzed. Abnormal SCV was detected in 7 cases (58.3%) and abnormal SNAP in 12 cases (100.0%). Both groups showed a trend that the abnormal rate of nerve action potentials (CMAP/SNAP) was higher than that of the corresponding nerve conduction velocity (MCV/SCV): In the SMN group, the abnormal rate of CMAP (92.3%) was higher than that of MCV (61.5%), and the abnormal rate of SNAP (84.6%) was higher than that of SCV (76.9%). In the SN group, the abnormal rate of SNAP (100.0%) was higher than that of SCV (58.3%). None of the above differences reached statistical significance (*P* > 0.05). Overall findings suggested that neuroelectrophysiological manifestations in both groups tended to be characterized by axonal involvement. Owing to the limited sample size, these results require further validation in larger scale prospective studies.

## 4 Discussion

A total of 92 patients diagnosed with pSS were enrolled in this study, among whom 25 cases (27%) presented with peripheral nervous system involvement. This finding is consistent with previous reports, which have indicated that the prevalence of neurological complications in patients with pSS ranges from 1.8% to 64% (14, 15). In the SMN and SN groups, female patients accounted for 80% and 100%, respectively, suggesting a higher prevalence of neurological complications in women compared to men (16). Consistent with prior research, the median age at onset for patients with SMN was 60 years, which was significantly higher than that of patients in the nPN group. Patients with pSS and peripheral nerve damage tend to have an older age at onset and diagnosis, and many present with neurological symptoms as the initial manifestation, making early recognition of pSS challenging (17–19).

In this study, patients in the SMN group exhibited lower positive rates of anti–SSA, anti–SSB, and RF compared with the nPN group. These serological features are consistent with observations reported in studies of small fiber neuropathy and SN associated with pSS (20, 21). Since this study did not examine the functions or cellular markers related to B–cell activation, the above results only describe correlations in serological indicators and cannot yet clarify the underlying mechanisms. We speculate that the development of SMN may not be mediated entirely by abnormal humoral immunity through the classical B–cell pathway, cellular immune-mediated inflammatory injury may also be involved in its pathogenesis.

Histopathological studies have suggested that T cells can infiltrate the vascular wall and cause endothelial cell damage, a process often accompanied by fibrinoid necrosis, vascular lumen occlusion, and ischemia (22); vasculitis has been reported in nerve biopsies of patients with symmetric SN and SMN (23). In addition to their direct cytotoxic effects, T cells also secrete proinflammatory cytokines such as interleukin-6 (IL-6) and interleukin-8 (IL-8), which form a microenvironmental regulatory network that may exacerbate nerve injury through paracrine signaling pathways (24). On the other hand, several studies have identified specific autoantibodies targeting neural tissues (14, 25). These antibodies bind to specific antigens in neural tissues to form immune complexes, which can trigger the production of toxic substances such as complements and directly attack nerve axons or myelin sheaths. Meanwhile, immune complexes may also deposit on the vascular walls of peripheral nerves, induce vasculitis, thereby resulting in neuronal dysfunction and impairing the nutritional metabolism and survival of neurons. For instance, Sallya et al. detected autoantibodies reacting with the muscarinic acetylcholine receptor 3 (M3R) in smooth muscle among patients with pSS. Widely expressed in salivary glands, lacrimal glands and autonomic nerve terminals, these autoantibodies can specifically interfere with M3R-mediated signaling pathways. Therefore, anti-M3R antibodies are considered crucial pathogenic factors underlying autonomic nervous dysfunction (26). In addition, Hsu et al. found that elevated serum levels of anti-β2 glycoprotein I antibodies and perinuclear anti-neutrophil cytoplasmic antibodies in pSS patients were positively correlated with the risk of neuropathy development, suggesting that these antibodies may participate in the pathological progression of pSS-related peripheral nervous system lesions by targeting vascular endothelial cells or neural tissue antigens (27).

Neurophysiological evaluation is essential for characterizing peripheral neuropathy (28). In the current study, both the SMN and SN groups showed a trend toward higher abnormality rates of nerve action potentials (CMAP/SNAP) than those of corresponding conduction velocities (MCV/SCV). Although these differences were not statistically significant, the neurophysiological pattern was suggestive of predominant axonal involvement, which is consistent with previous reports in pSS–related peripheral neuropathy (29). These findings suggest that immune-mediated injury in pSS may predominantly involve peripheral nerve axons.

SMN is a potentially disabling disorder, and optimal treatment strategies remain to be defined. Glucocorticoids are commonly used as first-line therapy, and 92.3% of patients in the SMN group received glucocorticoid treatment (30). The latest clinical recommendations from the European Alliance of Associations for Rheumatology (EULAR) indicate that, after excluding cryoglobulinemic vasculitis, glucocorticoids at a dose of 0.5~1mg/(kg·d) can be used for induction remission therapy in patients with multiple mononeuritis or vasculitis-associated axonal polyneuropathy. For severe peripheral neuropathy, the primary clinical goal is to control disease activity, adopting the lowest effective dose and shortest treatment course sufficient to maintain remission; when long-term maintenance therapy is required, combination treatment with immunosuppressive agents is recommended (31). On this basis, cyclophosphamide, as a potent immunosuppressant, is the preferred option for patients with critical conditions or an inadequate response to other medications (32). A number of existing case studies have confirmed that cyclophosphamide can significantly improve sensory and motor dysfunction in patients; improvements in nerve conduction velocity can be observed whether administered alone or in combination with intravenous immunoglobulin (33).

This study has several limitations. First, the sample size was relatively small due to the rarity of SMN, which may limit the statistical robustness of the findings. Second, this was a single-center retrospective study, which may be susceptible to selection bias and unmeasured confounding factors. Finally, the study lacked standardized evaluation of electrophysiological subtypes, vasculitic features, and cryoglobulinemia, thus failing to fully address the heterogeneity of pSS-related peripheral neuropathy, which limits the in-depth interpretation of pathophysiological mechanisms. Future multi–center prospective studies with detailed pathogenic stratification are warranted to validate these findings.

## 5 Conclusion

SMN is a common form of peripheral neurological involvement in patients with pSS. Patients with pSS–associated SMN tend to have an older onset age and lower positive rates of anti–SSA, anti–SSB, and RF, suggesting potentially distinct clinical and serological profile. Neurophysiological analysis reveals a trend toward predominant axonal involvement in both SMN and SN groups. The underlying immunological mechanisms remain to be fully elucidated. Further validation in large–scale prospective controlled studies is therefore warranted.

## Data Availability

The raw data supporting the conclusions of this article will be made available by the authors, without undue reservation.
